# The Coupled Physico-Biochemical Mechanisms of Skin Aging: From Dermal Fluid Dynamics and Mechanotransduction to Nutrient-Sensing Dysfunction

**DOI:** 10.7759/cureus.114591

**Published:** 2026-08-16

**Authors:** Zerong Zhang, Yong Liao

**Affiliations:** 1 Department of Dermatology, Bloomage Biotechnology Corporation Limited, Beijing, CHN; 2 Department of Medicine, Bloomage Biotechnology Corporation Limited, Beijing, CHN

**Keywords:** cell nutrient sensing, dermal fluid dynamics, extracellular matrix (ecm), mechanotransduction, skin aging

## Abstract

Traditional theories of early skin aging have established a robust foundation by elucidating the accumulation of molecular damage, such as inflammation, oxidative stress, epigenetic drift, and telomere shortening, and related extracellular matrix (ECM) changes. While these biochemical perspectives remain central to our understanding, increasing attention has been paid to the degeneration of the physical microenvironment of the dermal ECM and the concomitant dysfunction of core cellular functions during aging. This article aims to propose and substantiate an integrative framework for the early mechanisms of skin aging. Aging is not initiated by isolated molecular events but rather represents a tissue-level structural and functional degenerative process involving three coupled and progressively amplified dimensions: dermal fluid dynamics decay (characterized by decreased interstitial fluid pressure and impaired interstitial flow), mechanical unloading of fibroblasts (resulting from ECM fragmentation leading to diminished mechanical tension), and cellular nutrient-sensing dysfunction (involving dysregulation of the mechanistic target of rapamycin/AMP-activated protein kinase/sirtuin 1 pathways). Signaling axes, such as integrin-YAP/TAZ, constitute a core bridge linking these physical microenvironmental changes to intracellular biochemical responses. Based on this coupling framework, a progressively reinforced positive feedback loop model is further constructed, and its clinical implications for developing multitargeted, tissue-level skin aging intervention strategies are elaborated.

## Introduction and background

The clinical manifestations of skin aging encompass a series of morphological and functional changes, including wrinkle formation, skin laxity, loss of elasticity, and diminished repair capacity [1]. Classical theories of aging primarily explain the underlying mechanisms by focusing on the accumulation of molecular damage, which includes reactive oxygen species (ROS)-mediated oxidative stress, telomere shortening, DNA damage, loss of proteostasis, and the formation of advanced glycation end products (AGEs) [2-4]. In recent years, with advances in biomechanics, biorheology, and cellular metabolism, understanding the early events of aging from the perspective of the tissue’s extracellular matrix (ECM) physical microenvironment and cellular sensory functions has become a new research direction [5-7]. Early skin aging refers to a degenerative process occurring in individuals aged 19-30 years, characterized by the concurrent initiation of phenotypic and molecular alterations, followed by asynchronous and nonlinear progression [8-10]. While some researchers have proposed that the dermis functions as a sophisticated poroelastic biomaterial system [5], others have highlighted that the functional state of dermal fibroblasts is highly dependent on their mechano-coupling with the ECM [6]. Despite the wealth of studies investigating these individual mechanisms, the field currently lacks a comprehensive synthesis of the physico-biochemical coupling process. Therefore, this review aims to integrate contemporary research findings to provide a holistic perspective on skin aging.

The dermal layer of the skin is a dynamic, composite system consisting of the ECM, interstitial fluid, and functional cells. The maintenance of its function relies not only on the integrity of biochemical molecules but, more critically, on the physical homeostasis of the entire system and the cell’s precise perception of and response to the microenvironment [11]. The early progression of skin aging can be conceptualized as a coupled process in which the collapse of the dermal physical microenvironment and the dysfunction of cellular sensory mechanisms are intricately intertwined, forming positive and negative feedback loops, a conceptual framework we propose herein [12,13]. Specifically, this process can be deconstructed into three interrelated degenerative mechanisms: dermal fluid dynamics decay, mechanical unloading of fibroblasts, and cellular nutrient-sensing dysfunction.

Literature search strategy

This manuscript is a narrative review. We primarily surveyed peer-reviewed publications indexed in major biomedical databases, such as PubMed and Web of Science, focusing on original research and authoritative reviews related to skin aging mechanisms published within the last decade (2016-2026). The selection prioritized studies addressing the interplay between dermal ECM remodeling, cellular mechanotransduction, and metabolic regulation. To ensure scientific rigor, pivotal early landmark studies were retained, whereas mechanistically irrelevant studies were excluded to maintain focus on the core thesis.

## Review

The three core dimensions of early skin aging mechanisms

In research on early skin aging, we found that the decay of dermal fluid dynamics, mechanical unloading of fibroblasts, and cellular nutrient-sensing dysfunction exert a profound influence on the early progression of skin aging. These interrelated mechanisms establish a framework for understanding the tissue-level degenerative cascade.

Decay of Dermal Fluid Dynamics

Functioning as a poroelastic fluid system, the young dermis exhibits interstitial fluid pressure and directional flow, which are essential for homeostasis. Age-related degradation of hyaluronic acid (HA) and collagen culminates in compromised pressure and flow stasis, a pathological state termed “low-pressure collapse.”

The young dermis as a healthy poroelastic fluid system: The young, healthy dermis can be viewed as a sophisticated poroelastic biomechanical system at the biophysical level. Its hydration and permeability properties can be directly measured and modeled at the nanoscale, manifesting as solid matrix viscoelasticity dominating at lower frequencies (reflecting its viscoelastic nature), while fluid flow and extrusion within the pores become the primary energy dissipation mechanism at higher frequencies (highlighting its porosity) [5], with its material basis and functional analysis detailed in Table 1.

**Table 1:** Material basis and functional analysis of the dermal fluid dynamics system. ECM, extracellular matrix; GAGs, glycosaminoglycans; HA, hyaluronic acid; IFP, interstitial fluid pressure; MW, molecular weight Sources: [5-7]

Physical component	Representative substances	Biological function
Osmotic pressure builder	High-molecular-weight HA, chondroitin sulfate	Generates osmotic pressure through its hydrophilic nature, establishing an outward IFP that provides intrinsic volumetric support to the skin, constituting the biophysical basis of “plumpness.”
Fluid viscosity	GAGs (primarily high-MW HA), versican	Imparts viscous properties to interstitial fluid. Absorbs energy (damping effect) via fluid flow resistance upon skin compression, buffering mechanical shocks and protecting the fibrous skeleton.
Porosity	Small leucine-rich proteoglycans (e.g., decorin and lumican)	Regulates fiber diameter and spacing through steric hindrance, thereby determining collagen fiber spacing and alignment, which governs porosity.
Mechanical signal carrier	Free water, electrolytes (Na⁺/K⁺), fibronectin/laminin	During interstitial fluid flow through ECM pores, the resultant shear stress is transduced by mechanosensors (e.g., ion channels) on fibroblast surfaces or via fibronectin/laminin linkages to cell-surface integrin receptors, initiating intracellular signaling cascades.
Metabolic transport medium	Interstitial fluid, small nutrient molecules	Responsible for transporting amino acids and vitamins from capillaries to fibroblasts and removing metabolites via fluid exchange, maintaining microenvironmental homeostasis.
Space filling/lubrication	HA, versican	Fills spaces between collagen fiber bundles, reducing inter-fiber friction, providing hydrostatic pressure, and maintaining the flexibility of the ECM mechanical mesh.

Type I and III collagen fibers and the elastic fiber network are arranged regularly, forming the primary ECM skeleton that provides structural tension and elasticity [7]. The gel formed by highly hydrated glycosaminoglycans (GAGs), mainly HA, along with freely flowing interstitial fluid, fills the pores within the ECM fibrous framework [14].

This system maintains a tissue interstitial fluid pressure (IFP) slightly below atmospheric pressure, which has been empirically quantified by Wiig and Noddeland via measurement of IFP in normally hydrated human hand skin using micropipettes (tip diameter 2-4 μm), yielding a mean IFP of -3.1 mmHg (range: -5 to -0.5 mmHg) [15]. This pressure homeostasis arises from the dynamic balance between the osmotic swelling pressure generated by negatively charged GAGs and the mechanical constraint of the collagen network [16].

Anchored to this steady-state IFP, directional interstitial fluid flow propelled by trans-tissue compartmental pressure gradients confers endogenous “hydraulic support” to the skin, contributing to its fullness and turgor, but also performs critical physiological functions. These include the efficient delivery of nutrients and oxygen to cells and the removal of metabolites. Furthermore, the fluid shear stress generated by the flow itself acts as an important mechanical stimulus, directly regulating fibroblast gene expression and synthetic function [16-18].

Studies have shown that, in a stretched 3D collagen gel model seeded with human dermal fibroblasts under a perfusion regimen simulating the physiological interstitial flow velocity of native human dermis (~0.012 μm/s on average within the collagen matrix, up to 14.8 μm/s adjacent to the perfusion inlet, corresponding to a flow rate of 1 mL/d), interstitial flow upregulates the expression of hyaluronan synthase 2 and promotes HA synthesis, thereby contributing to ECM microenvironment homeostasis [19].

Tissue-level decay induced by aging: In the process of skin aging driven by intrinsic and extrinsic factors, significant qualitative and quantitative changes occur in the ECM: HA content decreases markedly, and high-molecular-weight HA is degraded into low-molecular-weight fragments, weakening its water retention and swelling pressure capacity [19]. Accumulating evidence indicates that diminished HA levels compromise tissue repair capacity [20]. Concurrent with these GAG alterations, the collagenous matrix undergoes molecular remodeling. Collagen fibers are degraded and fragmented by matrix metalloproteinases (MMPs; e.g., MMP-1 and MMP-3) and undergo abnormal cross-linking via AGEs, leading to a disordered, stiffened collagen network with reduced porosity [21,22]. Consequently, the biophysical properties of the dermal ECM poroelastic fluid system undergo alterations: the water-binding capacity of GAGs diminishes, reducing electrostatic swelling pressure; the disordered and fragmented collagen network disrupts pore structure, increasing resistance to interstitial fluid flow [16,23,24]. Consistent with predictions from established dermal poroelastic mechanobiological models, local IFP may decrease or even be lost, potentially leading to stagnant interstitial fluid flow and a hypothesized pathological state of “low-pressure collapse” [25]. With the persistence of this pathological state and progression of skin aging, lymphatic dysfunction (e.g., abnormalities in lymphatic endothelial cell junction proteins and failure of the smooth muscle pump) may emerge, which further exacerbates impaired tissue fluid drainage, leading to the accumulation of metabolites and inflammatory mediators, thereby fostering a microenvironment detrimental to tissue homeostasis and accelerating aging [26]. Fibroblasts consequently lose their healthy fluid mechanical microenvironment, entering a state of functional suppression characterized by insufficient nutrient supply and impaired mechanosignaling.

Mechanical Unloading of Fibroblasts

Age-related ECM degradation engenders mechanical unloading of fibroblasts, compromising adhesion and attenuating collagen synthesis. This cascade induces a catabolic phenotype, thereby precipitating skin laxity and wrinkle formation.

The ECM provides critical mechanical scaffolding for cells: The functional state of dermal fibroblasts is highly dependent on their mechanical coupling with the ECM [27-29]. Through transmembrane adhesion receptors such as integrins, cells stably adhere to a dense, continuous collagen fiber network with appropriate stiffness [6]. This connection enables the cytoskeleton (primarily composed of actin stress fibers) to establish intrinsic tension, maintaining the cell in a suitably spread morphology and subjecting it to continuous physiological mechanical stimulation [27]. Integrin α11β1, a collagen-binding receptor preferentially enriched in dermal fibroblasts, functions as the principal mechanosensor governing cell-ECM interplay [28]. This receptor employs its transmembrane domains to physically couple the dense, continuous collagenous matrix to the intracellular actin cytoskeleton, thereby nucleating stable focal adhesions (FAs) [29]. This mechanical state serves as a key physical foundation for maintaining cell viability, a youthful phenotype, and anabolic activity. Studies have confirmed that fibroblasts cultured on substrates with physiological stiffness exhibit significantly higher collagen synthesis activity compared with those cultured on soft substrates [30].

Mechanical signaling imbalance triggered by ECM degradation: During the process of skin aging, multiple factors converge to induce MMP-mediated degradation and fragmentation of the collagen network [31]. This process results not only in structural rupture and disarray of collagen fibrils but also in pathological remodeling of their biophysical properties, specifically, a marked increase in fibrillar surface roughness coupled with concurrent elevations in stiffness and hardness of the fiber bundles [32]. Collectively, these alterations directly compromise the structural continuity and mechanical integrity of the ECM. Consequently, fibroblasts lose the continuous mechanical scaffold essential for attachment and spreading, leading to impaired cell-matrix adhesion formation, interruption of integrin-mediated mechanotransduction, and a significant reduction in the mechanical tension borne by the cells [33]. The cell morphology shifts from an extended spindle shape to a rounded or poorly spread state, accompanied by cytoskeletal reorganization and a marked decrease in surface area and effective interaction area with the ECM [34]. This state of “mechanical unloading” transmits aberrant signals intracellularly via pathways such as integrin-FAK-Rho/ROCK, resulting in a significant decline in the fibroblast’s ability to synthesize type I/III collagen and elastin [12]. Compensatorily, cells may secrete more MMPs and senescence-associated secretory phenotype (SASP) factors [13], thereby forming a vicious cycle: “ECM degeneration → mechanical unloading → suppressed synthesis/enhanced degradation → further ECM degeneration.” This process constitutes a core cellular mechanism underlying the loss of skin elasticity, laxity, and wrinkle formation.

Cellular Nutrient-Sensing Dysfunction

Age-related dysfunction of nutrient-sensing pathways (mechanistic target of rapamycin complex 1 (mTORC1), AMP-activated protein kinase (AMPK), and sirtuin 1 (SIRT1)) culminates in a state of “synthetic stasis.” Cells exhibit impaired biosynthetic conversion of nutrients into ECM components, thereby exacerbating functional decline in aged skin.

Core regulatory hubs of the nutrient-sensing system: Cells precisely sense and regulate the uptake, storage, and biological utilization of nutrients through a series of evolutionarily conserved nutrient-sensing pathways [35]. The mTORC1 acts as a key anabolic regulatory hub, sensing signals such as amino acids and growth factors [36]; its activation potently drives ribosome biogenesis, protein (including collagen) synthesis, and lipid synthesis [37]. AMPK is the central sensor of cellular energy status (with the AMP/ATP ratio as a core indicator), which is activated under energy deficit to restore homeostasis by inhibiting anabolism and promoting catabolism (e.g., activating autophagy) [38]. SIRT1 is an NAD+-dependent deacetylase involved in metabolic regulation, stress response, and the maintenance of genomic stability [39]. These pathways interact extensively, collectively forming the core network of cellular metabolic regulation.

Age-related dysfunction in nutrient perception: Prior studies have formally categorized deregulated nutrient sensing as a primary hallmark driving organismal aging. When nutrient availability is excessive or imbalanced, intracellular nutrient-sensing pathways, principally encompassing insulin/IGF-1 signaling, mTOR, AMPK, and sirtuins, transition into a state of persistent dysregulation, which directly accelerates organismal aging [40]. During the process of aging, even when systemic nutrient circulation is adequate, dermal fibroblasts often develop nutrient-sensing dysfunction [41,42]. Specific manifestations include reduced responsiveness or abnormal sustained activation of the mTORC1 pathway to nutrient stimuli, leading to an imbalance between anabolic and autophagic processes [43]; decreased AMPK activity, weakening the cell’s ability to sense and respond to energy crises (e.g., elevated AMP/ATP ratio) [44]; and reduced expression and enzymatic activity of SIRT1, resulting in impaired regulation of metabolism and stress pathways dependent on deacetylation [45]. The consequence is that cells enter a state of “synthetic stasis,” a condition of functional nutrient deficiency wherein, despite a relatively nutrient-rich environment, cells are unable to efficiently convert substrates like amino acids and glucose into new ECM components [46]. Compounding this paradox is age-related mitochondrial dysfunction leading to insufficient ATP production, which further exacerbates the cellular energy and nutrient supply crisis [47]. This intracellular impairment in perceiving and utilizing metabolites is a fundamental reason for the diminished repair, regeneration, and ECM synthesis capacity of aged skin [48].

Integration of Core Signaling Pathways: The Bridge Linking Microscopic Instability to Macroscopic Aging

The integrin-YAP/TAZ axis and its bidirectional crosstalk with nutrient-sensing pathways serve as pivotal conduits linking microscopic ECM perturbations to macroscopic aging phenotypes. Elucidation of these axes clarifies how biophysical perturbations are transduced into cellular dysfunction.

The integrin-YAP/TAZ axis: a central pathway connecting the mechanical microenvironment to cellular function: The integrin family serves as the primary receptor for cells to perceive the physical and biochemical properties of the ECM. They not only specifically recognize ECM ligands (such as collagen and fibronectin) but also sense matrix stiffness, topology, and fluid mechanical stimuli [48]. Integrin α11β1 is the predominant collagen-binding receptor in human dermal fibroblasts [29,49]. In in vitro aging models of senescent dermal fibroblasts, the mRNA expression of ITGA11, the core gene encoding integrin α11, and α11 protein levels are significantly diminished. This reduction compromises the cell-collagen interaction and adhesive capacity of senescent cells, along with their overall cell spreading and morphological characteristics [25]. When the ECM becomes degraded, fragmented, or altered in stiffness due to aging, the assembly and maturation of integrin-mediated FAs are impeded, leading to aberrant downstream signal transduction [50]. YAP and its homolog, TAZ, are core downstream effectors of mechanical signal transduction, acting as the "master switches" of the cellular mechanical state [51]. Their subcellular localization and transcriptional activity are precisely regulated by cytoskeletal tension, cell morphology, cell density, and ECM stiffness [52]. Under healthy mechanical loading (with well-spread cells and high cytoskeletal tension), YAP/TAZ are activated and translocate to the nucleus, where they bind to transcription factors like TEAD to initiate the expression of a series of target genes, including connective tissue growth factor, cyclin D1, and the collagen type I alpha 1 chain, thereby maintaining the synthetic, proliferative, and anti-apoptotic state of fibroblasts [53]. Studies indicate that nuclear localization and transcriptional activity of YAP/TAZ are significantly reduced in fibroblasts from aged or photoaged skin. This decline in activity not only directly leads to reduced synthesis of ECM components like collagen but may also relieve the inhibition on innate immune pathways such as cGAS-STING, exacerbating chronic low-grade inflammation (i.e., inflammaging), thereby further disrupting skin ECM microenvironment homeostasis [54].

Crosstalk between mechanical and nutrient signaling: interaction between mTOR and YAP/TAZ: Mechanotransduction pathways and nutrient-sensing pathways do not operate in isolation; they are tightly coupled through bidirectional crosstalk between YAP/TAZ and the mTORC1 pathway [18]. On one hand, activated YAP/TAZ can transcriptionally upregulate amino acid transporters and key components of the mTORC1 pathway, thereby promoting mTORC1 activation [55]. On the other hand, mTORC1, by regulating processes such as cell growth and ribosome biogenesis, affects the overall biosynthetic capacity of the cell, thereby indirectly supporting the anabolic programs driven by YAP/TAZ [56]. More importantly, both share upstream regulatory factors, such as Rho GTPases [55,57]. Therefore, nutrient sufficiency (activating mTORC1) and intact mechanical signaling (activating YAP/TAZ) mutually reinforce each other, collectively maintaining normal fibroblast function. Conversely, the loss of either signal (e.g., nutrient-sensing dysfunction or mechanical unloading) can impair the function of the other, leading to a decline in cellular function.

Construction of the coupling model and its clinical implications

We propose an integrative coupling model delineating self-amplifying feedback loops among the three core dimensions. This framework advocates multi-target intervention strategies to arrest the aging cycle, underscoring the immense potential of coordinated approaches for anti-aging.

Constructing the Coupling Model: A Feedback Loop of Three Dimensions

Based on the aforementioned discussion, we construct a coupling model for the early stages of skin aging. This model illustrates how dermal fluid dynamics decay, mechanical unloading of fibroblasts, and cellular nutrient-sensing dysfunction form a progressively amplified, self-reinforcing feedback loop.

Initiation stage: Established research demonstrates that intrinsic aging or extrinsic damage (e.g., ultraviolet radiation) causes initial degradation of the ECM, manifested as reduced HA content and early fragmentation of the collagen network. Consistent with the proposed conceptual framework, this may simultaneously trigger the onset of fluid dynamics decay (beginning decline in IFP and impaired interstitial flow) and mechanical unloading (reduction in effective cell-ECM anchoring points) [58].

First-stage progression: Conclusive experimental evidence demonstrates that mechanical unloading causes fibroblast morphology to shift from a spread to a rounded state, with decreased YAP/TAZ transcriptional activity and reduced HA and collagen synthesis capacity. Concurrently, aberrant mechanical signals activate inflammation-related pathways like NF-κB via integrins, prompting cells to secrete more MMPs and inflammatory factors, thereby accelerating ECM destruction and forming a local feedback loop of “ECM degradation → inhibition of cellular function” [50-52].

Second-stage progression: The degenerated ECM (with reduced porosity and further loss of HA) theoretically further impedes interstitial fluid flow, exacerbating fluid dynamics decay. The efficiency of local oxygen and nutrient delivery declines significantly, and metabolic waste accumulates. Age-related decline in lymphatic vessel function leads to impaired tissue fluid drainage, worsening tissue edema and fostering a pro-inflammatory microenvironment [13,19,26].

Stabilization and propagation stage: Consistent with the aforementioned conceptual framework, impaired nutrient delivery and persistently dysregulated mechanical signals collectively act on the cell interior and may cause profound dysregulation of core nutrient-sensing pathways (mTOR/AMPK/SIRT1). Cells may progressively lose the ability to coordinate anabolic and catabolic metabolism, entering a state of “synthetic stasis” that is difficult to reverse, and, through the secretion of SASP factors that influence neighboring cells, the senescent phenotype becomes entrenched and spreads within the tissue [56].

Implications for Anti-Aging Strategies

This coupling model offers a potential, multitarget conceptual framework that may inform the development of more effective skin aging intervention strategies. Therefore, rational anti-aging approaches might benefit from moving beyond single-molecule or single-pathway targeting toward coordinated interventions across the aforementioned three core dimensions, pending further preclinical and clinical validation.

Particularly in the early stages of aging, it is crucial to restore and maintain dermal fluid pressure (IFP) and interstitial flow, for example, through exogenous supplementation of high-molecular-weight HA or the use of ingredients promoting endogenous HA synthesis (e.g., N-acetylglucosamine) [21], and supplementing with collagen, elastin, and proteoglycans to sustain the structural support of the ECM. Mosteirin et al. conducted a systematic review and meta-analysis comprising 11 clinical studies (including four randomized controlled trials and seven observational studies) to evaluate the efficacy and safety of injectable HA supplemented with amino acids for facial rejuvenation in adults. Pooled results demonstrated that, compared with conventional HA fillers or placebo, amino acid-supplemented HA preparations achieved superior aesthetic outcomes, enhanced structural integrity, and more durable volumetric effects [59].

Further corroborating these pooled findings, a multicenter, randomized controlled trial conducted by Yang et al. demonstrated that mesotherapeutic injection of an HA-based complex solution supplemented with proline and other amino acids significantly ameliorated clinical manifestations of facial skin aging, achieving a response rate of 91.87% at the three-month follow-up and demonstrating a favorable safety profile. This evidence substantiates the proposed framework, indicating that the synergistic improvement of ECM biomechanical properties and nutrient sufficiency constitutes a viable strategy to attenuate skin aging phenotypes [60].

Simultaneously, protecting ECM integrity and providing appropriate mechanical loading are essential. Synthesizing available evidence, potentially efficacious strategies include using HA with low cross-linking and specific viscoelasticity, inhibiting MMP activity (e.g., retinoids, specific peptides) [61], counteracting nonenzymatic collagen glycation (e.g., carnosine) [56], and utilizing minimally invasive techniques like microneedling, fractional lasers, or radiofrequency to induce controlled post-wound repair, thereby remodeling a healthy ECM structure and reactivating the YAP/TAZ pathway by reintroducing mechanical stimulation (e.g., poly-L-lactic acid, poly-D,L-lactic acid, and calcium hydroxyapatite). In this process, schizophyllan and similar ingredients can promote ECM synthesis [62], while salidroside and similar ingredients protect ECM from oxidative damage [63].

Furthermore, improving cellular nutrient perception and utilization efficiency is vital. Synthesis of extant evidence suggests that potential strategies may involve applying ingredients that activate AMPK (e.g., resveratrol), inhibit overactive mTORC1 (e.g., rapamycin derivatives), or activate SIRT1 (e.g., β-nicotinamide mononucleotide, pyrroloquinoline quinone, and ergothioneine) to reset cellular metabolic sensing [64] and directly supplementing with readily utilizable nutrients that promote ECM synthesis and sustain dermal metabolic homeostasis (e.g., amino acids, human milk oligosaccharides, polydeoxyribonucleotide, sialic acid, and fucose) to support anabolism [65-67].

Based on the above framework, the combined application of these multidimensional strategies may theoretically yield synergistic effects, more effectively disrupting the vicious cycle of aging. However, as this framework comprises substantial theoretical postulates, rigorous clinical trials are urgently warranted to validate its scientific robustness and facilitate the translation of this conceptual model from theoretical conception to clinical practice.

Discussion

This review advocates a conceptual shift from the conventional view of skin aging as isolated molecular damage toward a recognition of it as a coupled, tissue-level disorder driven by the dynamic interplay of dermal fluid dynamics, fibroblast mechanobiology, and cellular nutrient sensing [5-57]. Building on a systematic synthesis of pertinent literature and integrating established findings on fibroblast mechanotransduction mechanisms and canonical cellular nutrient-sensing pathways, we provisionally propose the novel theory of dermal fluid dynamics decay. Critically, by constructing this integrative three-dimensional framework, which synthesizes well-documented mechanobiological and metabolic regulatory mechanisms while introducing the previously unrecognized contribution of dermal fluid dynamics decay, we address a critical lacuna in classical theories, which have historically prioritized biochemical cascades (e.g., ROS and AGEs) while overlooking the instrumental role of the physical microenvironment in initiating and amplifying aging phenotypes.

A central tenet of this framework is the identification of the integrin-YAP/TAZ-mTOR axis as a pivotal signaling nexus, mechanistically bridging ECM physical perturbations to intracellular metabolic dysregulation [21,55-57]. Specifically, ECM fragmentation imposes a dual pathogenic insult: it attenuates mechanical tension while concurrently compromising interstitial fluid flow. This tandem disruption suppresses fibroblast anabolism and promotes catabolic signaling (e.g., MMP secretion) [21,22], thereby challenging prior models that relegate “structural degradation” and “functional decline” to parallel, rather than causally linked, processes [5-13].

While the proposed integrative coupling model provides a novel conceptual framework for deciphering early skin aging, several limitations warrant careful consideration. First, core mechanistic inferences, including the ECM fragmentation-initiated cascade and integrin-YAP/TAZ-mTOR crosstalk, are largely extrapolated from noncutaneous tissues, in vitro fibroblast models, or theoretical poroelastic frameworks, lacking direct validation in longitudinal aged human skin samples. Second, scarce longitudinal human data preclude defining the temporal hierarchy and causality among the three core dimensions, as most supporting evidence derives from cross-sectional observational studies. Third, clinical translatability remains unproven: no head-to-head comparative trials demonstrate that multidimensional interventions outperform established single-target or combination therapies, and the synergistic benefits of proposed strategies await rigorous prospective validation.

Prospective investigations should prioritize the quantitative delineation of the dynamic evolutionary relationships among these dimensions. A critical endeavor involves the discovery and validation of noninvasive or minimally invasive biomarkers reflective of early coupled dysfunction, such as specific ECM degradation fragments, subtle perturbations in IFP, or alterations in core ECM constituents. Moreover, leveraging this coupling model, multitarget synergistic interventions and combination treatment regimens should be rationally designed and rigorously validated. Through targeted modulation of these coupled physico-biochemical mechanisms, we anticipate forging new pathways for the development of precise and effective comprehensive management strategies for early skin aging.

## Conclusions

This article systematically elaborates that the early progression of skin aging should not be construed as originating solely from isolated molecular damage events. Rather, based on a synthesis of current evidence, we propose an integrative conceptual framework wherein early skin aging represents a coupled, progressively amplified, tissue-level functional degeneration. Conceptually, this progression primarily involves three interrelated dimensions: dermal fluid dynamics decay, mechanical unloading of fibroblasts, and cellular nutrient-sensing dysfunction. Core signaling axes, particularly the integrin-YAP/TAZ-mTOR axis, are proposed as critical mechanistic hubs connecting the deterioration of the ECM physical microenvironment to intracellular metabolic dysregulation. It is important to emphasize that while this framework provides a coherent synthesis of existing literature, the precise hierarchical sequence of these interconnected events requires further validation in longitudinal human studies and remains a theoretical model rather than a definitively established paradigm of human skin aging.
